# Proline Priming Enhances Seed Vigour and Biochemical Attributes of Rice (*Oryza sativa* L.) during Germination

**DOI:** 10.21315/tlsr2024.35.3.7

**Published:** 2024-10-07

**Authors:** Cloee Kher Yan Kong, Rattanak Sambath Lee, Kamariah Hasan, Clement Kiing Fook Wong, Chui Yao Teh

**Affiliations:** 1 School of Applied Sciences, Faculty of Integrated Life Sciences, Quest International University, No. 227, Jalan Raja Permaisuri Bainun, 30250 Ipoh, Perak, Malaysia; 2 School of Postgraduate Studies, Research and Internalisation (SPRINT), Faculty of Integrated Life Sciences, Quest International University, No. 227, Jalan Raja Permaisuri Bainun, 30250 Ipoh, Perak, Malaysia; 3 Department of Agricultural and Food Science, Faculty of Science, Universiti Tunku Abdul Rahman, Jalan Universiti, Bandar Barat, 31900 Kampar, Perak, Malaysia; 4 Centre for Agriculture and Food Research, Universiti Tunku Abdul Rahman, Jalan Universiti, Bandar Barat, 31900 Kampar, Perak, Malaysia

**Keywords:** Biochemical Changes, Germination, Priming, Proline, Seed Vigour, Perubahan Biokimia, Percambahan, Prima, Prolin, Vigor Benih

## Abstract

Seed vigour is a desirable trait especially for direct seeded rice (DSR) cultivation. Seeds with high vigour could improve seed germination, support seedlings in competing with weeds for water and nutrients, and improving seedling establishment throughout the early stages of crop growth. The success of DSR system which account for more 25% of world cultivation areas is highly dependent on the seed vigour and seedling establishment. Seed priming is a promising technique to improve seed vigour. Proline is an amino acid that has been well studied for its roles in plants under different environmental stress conditions. Nevertheless, the effect of proline as a seed priming agent in improving seed vigour in rice remain elusive. In this research, the effect of 24 h of proline priming at various concentrations (0 mM, 1 mM, 2 mM, 10 mM and 20 mM) on rice seed vigour, amylase activity, and total soluble sugar (TSS) content of a Malaysia *indica* rice variety, MR269 was investigated. Results showed that seeds primed with lower concentration of proline (0 mM, 1 mM and 2 mM) had better germination responses while priming at high concentrations (10 mM and 20 mM) reduced seed germination. Among the concentration tested, priming with 1 mM proline enhanced seed vigour with significantly higher germination percentage (GP), germination rate index (GRI) and seedling vigour index (SVI). In addition, proline primed seeds also exhibited increased amylase activity and TSS content as compared to unprimed seeds. However, priming seed with 20 mM proline was detrimental to the seed vigour and seedling growth whereby lower GP, GRI and SVI and higher mean germination time (MGT) were observed. In short, this study shows that proline could be a potential seed priming agent to improve seed vigour in rice.

## Abstract (fr)

**Abstrak:**

Vigor benih merupakan sifat yang diingini dalam penanaman padi terutamanya dalam sistem pembenihan langsung (DSR). Benih dengan vigor yang tinggi boleh meningkatkan kadar percambahan, menyokong anak benih untuk bersaing dengan rumpai untuk mendapatkan air dan nutrien, serta meningkatkan pertumbuhan anak benih pada peringkat awal tanaman. Kejayaan sistem DSR yang merangkumi lebih 25% kawasan penanaman dunia sangat bergantung kepada vigor benih dan penubuhan anak benih. Rawatan biji benih merupakan satu teknik yang mampu meningkatkan vigor benih. Prolin merupakan asid amino yang mempunyai peranan yang penting dalam tumbuhan di bawah keadaan tekanan persekitaran yang berbeza. Namun begitu, kesan prolin sebagai agen rawatan benih dalam meningkatkan vigor benih masih lagi tidak jelas. Dalam penyelidikan ini, kesan larutan prolin pada kepekatan yang berlainan (0 mM, 1 mM, 2 mM, 10 mM dan 20 mM) ke atas vigor benih padi, aktiviti amilase, dan jumlah kandungan gula larut (TSS) telah dikaji. Keputusan menunjukkan benih yang dirawat dengan kepekatan prolin yang rendah (0 mM, 1 mM, and 2 mM) menunjukkan kadar percambahan yang lebih baik manakala rawatan pada kepekatan yang tinggi (10 mM and 20 mM) mengurangkan kadar percambahan. Rawatan dengan 1 mM larutan prolin didapati dapat meningkatkan peratusan percambahan (GP), indeks kadar percambahan (GRI) dan indeks vigor benih (SVI) berbanding rawatan yang lain. Selain daripada itu, biji benih yang dirawat juga menunjukkan peningkatan aktiviti amilase dan TSS berbanding dengan biji benih tidak prima. Walau bagaimanapun, rawatan benih dengan larutan prolin pada kepekatan 20 mM didapati memudaratkan vigor benih di mana GP, GRI dan SVI yang lebih rendah dan masa percambahan minimum (MGT) yang lebih tinggi diperhatikan. Kesimpulannya, kajian ini menunjukkan bahawa prolin berpotensi untuk dijadikan agen rawatan benih untuk meningkatkan vigor benih padi.

**Highlights**
- Priming with 1 mM proline significantly enhanced MR269 seed vigour with higher germination percentage (GP), germination rate index (GRI) and seedling vigour index (SVI).
- Proline primed seeds also exhibited increased amylase activity and total soluble sugar (TSS) content as compared to unprimed seeds.
- Priming with 20 mM proline was detrimental to the seed vigour and seedling growth whereby lower GP, GRI, SVI and higher mean germination time(MGT) were observed.

## INTRODUCTION

Rice is an important element in the daily meals of people from Southeast Asian countries, including Malaysia (Rahim *et al*. 2017). Transplanting and direct seeding are the two common rice cultivation methods in Asia (Kumar & Ladha 2011). Lately, direct-seeded rice (DSR) system which involved growing rice from seeds sowed in the field rather than seedlings transplanted from a nursery has gained attention due to its low input demand (Kumar & Ladha 2011; Kaur 2020). Many farmers in the Philippines, Malaysia, Thailand and India have changed to DSR because of the introduction of early-maturing cultivars and improved nutrient management techniques, as well as increasing availability of chemical weed control measures (Farooq *et al*. 2011). More than 25% of the world rice cultivation area has adopted DSR system as the principal rice establishment method (Kumar & Ladha 2011; Panda *et al*. 2021). The DSR has quickly gained popularity as it produces higher yield, expedites the sowing process, provides better water and labour use efficiency, and decreases greenhouse gases emission (Panda *et al*. 2021) as compared to manual transplanting. Nevertheless, the success of DSR is highly dependent on the seed vigour and establishment of the seedlings during early growth stage.150

Seed vigour is a desirable but complex feature in rice. It helps seedlings in competing with weeds for water and nutrients, as well as improving seedling establishment throughout the early stages of crop development (Rebolledo *et al*. 2012; Anandan *et al*. 2020). The important parameters for good crop establishment are quick and uniform emergence as well as higher biomass in the early stages of crop growth (Mahender *et al*. 2015). These agronomic parameters are highly associated with seed vigour. In addition, the levels of glucose, amylase, growth hormones, antioxidant enzymes and ascorbic acid were reported to be associated with seed vigour (Mahender *et al*. 2015). Tabassum *et al*. (2017) reported that the amylase activity and soluble sugar content during germination are important factors in ensuring consistent emergence in seedlings. Guzman *et al*. (2017) reported a peak in starch degradation in germinating rice seeds after an increase in α-amylase levels. The elevated levels of α-amylase release glucose that would be transported to the embryo to enhance seed germination and radicle growth (Guzman *et al*. 2017). Rebolledo *et al*. (2012) reported that starch content was a useful marker for detecting early vigour in rice. Rice with low seedling vigour usually had a lower starch content and reduced plant growth.

Seed priming is an effective technique to improve seed vigour and germination which in turn produces sturdier plants and high yield (Singh *et al*. 2015). Priming works by stimulating internal biological processes during the pregermination period to prepare the seed better for the actual germination process (Mustafa *et al*. 2017). The application of seed priming has been shown to enhance crop tolerance under various biotic and abiotic stress conditions, promote seedling development, and increase yield in various agriculture crops (Rajendra *et al*. 2016). In rice, seed priming boosted seed germination, sprouting, growth and yield (Matsushima & Sakagami 2013; Tabassum *et al*. 2017). For instance, better growth of seedling in terms of plumule length, root length, seedling vigour, seedling biomass and germination index were observed in the primed seeds as compared to the unprimed seeds (Ali *et al*. 2020). Some of the priming techniques includes the osmo-priming, nutrient priming, chemical priming, bio-priming, hydro-priming and nano priming (Waqas *et al*. 2019).

Proline is a multifunctional amino acid that is able to protect plants under different environmental stress conditions (Cao *et al*. 2020). Recent research has suggested that exogenous application of proline could alleviate or reduce the detrimental effects caused by various abiotic stresses such as salinity stress (El Moukhtari *et al*. 2020), drought and heat stress (Hanif *et al*. 2021). Teh *et al*. (2016) reported the proline supplementation can effectively ameliorate damage caused by salinity stress in rice with improved plant height, fresh weight, and increased internal proline and chlorophyl contents. In addition, the authors’ preliminary studies have shown that proline supplementation results in increased plant weight, root size and numbers during phosphorus deficiency.

Despite the promising roles of proline in plant stress tolerance, limited study has been conducted to examine the potential of proline as a seed priming agent to improve seed vigour in rice. In this study, the effect of different concentrations of proline priming on rice seed vigour will be investigated. In addition, the biochemical changes in the rice seeds such amylase activity and total soluble sugar (TSS) at different time points after germination will also be investigated to provide a better understanding on the effect of proline on the biochemical parameters.

## MATERIALS AND METHODS

### Plant Materials

The plant material used in this study was matured rice seeds of a Malaysian *indica* rice cultivar, MR269 obtained from the Department of Agriculture, Sungai Burong, Selangor. This rice cultivar is a high yielding variety with its potential yield reaching up to 10 t ha^−1^ (Hashim *et al*. 2022). Besides, MR269 is also resistant to leaf blast and moderately resistant to panicle blast (Misman & Zakaria 2019).

### Seed Priming Treatment and Germination

Seed priming was conducted as described by Singh *et al*. (2018) with some modifications. Prior to priming, the rice seeds were surface sterilised using 70% (v/v) ethanol for 60 sec and 20% (v/v) commercial Clorox (containing 5% NaOCl) for 30 min to remove potential contaminants. The sterilised seeds were further rinsed three times using distilled water and blotted dry with filter paper. For proline priming, the sterilised seeds were immersed in proline solution with different concentrations from 0 mM, 1 mM, 2 mM, 10 mM and 20 mM for 24 h. Seeds not soaked in any solution served as control. Seed germination was performed according to Liu *et al*. (2018) with some modifications. Briefly, a piece of Whatman no. 1 filter paper was placed on a 90 mm Petri dish where 6 mL of distilled water was added (Fogliatto *et al*. 2019). Subsequently, the primed and unprimed seeds were placed onto the Petri dish. Seeds were incubated at 28°C for one week for germination. There were three replicates for each treatment and 60 seeds were used for each treatment. Seedlings with root length of ≥ 1 cm and shoot length ≥ 0.5 cm were considered germinated (Liu *et al*. 2018).

### Determination of Seed Vigour and Germination Capacity

To compare the seed vigour and germination capacity of the primed and unprimed seeds, the germination percentage (GP), mean germination time (MGT), germination rate index (GRI) and seedling vigour index (SVI) were determined using the following equations (Shatpathy *et al*. 2018; Zhang *et al*. 2023).


$$\text{GP}=\frac{\text{Number of seeds germinated on the }3\text{rd day}}{\text{Total number of seeds}}\times 100\%$$

$\text{MGT}=\frac{\Sigma nD}{\Sigma n}$, where *n* is the number of germinated seeds, D indicates $\text{GR}=\frac{G_{1}}{T_{1}}+\frac{G_{2}}{T_{2}}+\ldots \ldots +\frac{G_{n}}{T_{n}}$, where *G* is the number of germinated seeds in a particular day, *T* represents the time period (in days). SVI = (root length + shoot length) × GP number of days.

### Biochemical Responses of Rice Seed during Seed Germination

#### Extraction of crude amylase

The crude amylase was extracted according to Liu *et al*. (2018) with slight modifications. Briefly, five germinated seeds at 0 day, 4 days, 8 days and 12 days of germination were collected, ground and mixed with 10 mL of chilled distilled water for enzyme extraction. The mixture was incubated in an ice bath at 4°C for 10 mins with occasional agitation followed by centrifugation at 10,000 × g for 10 mins at 4°C. The clear supernatant was harvested and used as the crude extract for amylase and TSS assays.

#### Qualitative estimation of amylase activity

Qualitative assay of amylase activity was performed using the starch agar plate method as described by Liu *et al*. (2018). Briefly, the seed embryo was cut into half and the endosperms was surface sterilised in 20% (v/v) commercial Clorox (containing 5% NaOCl) for 20 mins, rinsed with distilled water for six times and soaked in distilled water for 24 h at 26°C (Yin *et al*. 2011). The halved seeds were placed on 2% (w/v) starch agar plate, pH 5.3 (0.2% soluble starch, 10 mM sodium acetate and 2 mM CaCl_2_). The plates were then incubated at 28°C for 48 h. After incubation, the plates were stained with 2 mL of I_2_/KI solution (2.8 mM I_2_, 43.4 mM KI in 0.2 N HCl) for 5 mins until the agar was turn into blue-purple colour. The presence of amylase was indicated with the formation of clear zone around the seeds.

#### Quantitative assay of amylase activity

The quantitative amylase activity was conducted using 3,5-dinitrosalicylic acid (DNSA) method as described by Li *et al*. (2019) with slight modifications. The crude extract derived was heated for 15 mins at 70°C. Subsequently, 1 mL of crude extract was mixed with 1 mL of 1% (w/v) soluble starch dissolved in a citric acid buffer (pH 5.6). The mixture was then placed in a water bath for another 5 mins at 40°C. Subsequently, 2 mL of DNSA reagent (1 g of DNSA, 30 g of sodium potassium tartrate and 20 mL of 2N sodium hydroxide) was added into the mixture (Kamtekar *et al*. 2014). The mixture was boiled for 5 mins. The mixture was cooled to room temperature under tap water. The colour of reducing sugar was estimated using a UV-Vis spectrophotometer (UV/VIS T60, PG Instruments Limited, Leicestershire, UK) at 540 nm. A standard curve was generated using maltose as reducing sugar standard to calculate the amylase activity. One unit of α-amylase activity was defined as the amount of enzyme that produced 1 μM of maltose per minute under the enzyme activity conditions.

#### Determination of TSS content

TSS content was determined using the phenol sulfuric method as described by Sheteiwy *et al*. (2017) with slight modifications. Briefly, 0.5 mL of the crude extract was diluted by adding 1.5 mL distilled water. Then, 1 mL of 5% (w/v) phenol and 5 mL of concentrated sulfuric acid were added into the mixture. The mixture was measured using a UV-Vis spectrophotometer at 485 nm and the amount of TSS was determined from a glucose standard curve and expressed as mg/g fresh weight (FW).

### Statistical Analysis

All the data collected were analysed using one-way ANOVA test via Statistical Package for the Social Sciences (SPSS) version 25.0 (IBM Corp., Armonk, NY, US). Duncan’s New Multiple Range Test (DMRT) was used to compare the mean values of each treatment at 5% significance level.

## RESULTS

### Seed Vigour and Germination Capacity

**Figure 1:**
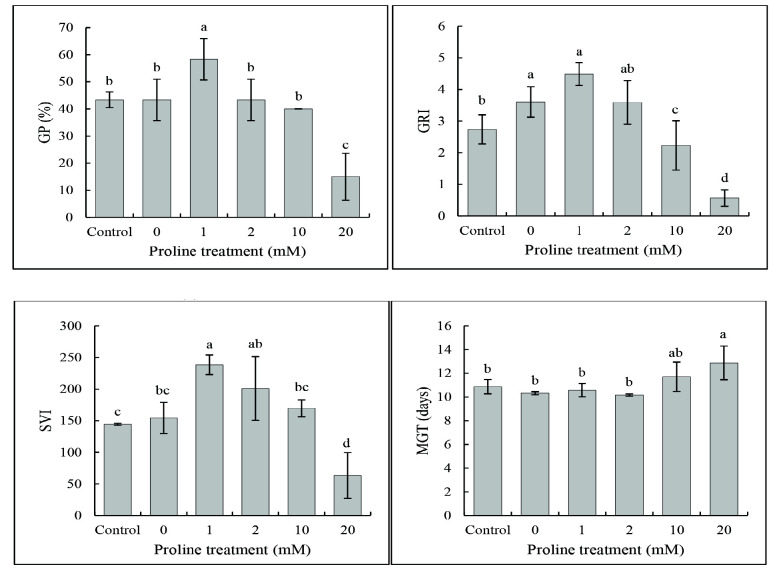
Effect of proline priming on seed vigour and germination capacity: (a) GP; (b) GRI; (c) SVI; and (d) MGT. *Notes*: Bar indicates mean standard deviation of three replicates. Different lowercase letters above the bars indicate their relative significance at *p* < 0.05 probability value.

The germination of proline-primed and unprimed seeds was monitored for one week. Results showed that seeds primed with lower concentration of proline (0 mM, 1 mM and 2 mM) had better germination responses while priming at high concentrations (10 mM and 20 mM) reduced seed germination (Fig. 1). The highest GP was recorded in 1 mM proline (58.3%) and the lowest was recorded in 20 mM proline concentration (15.0%). Meanwhile, the GP of seeds primed with 0 mM (43.3%), 2 mM (43.3%) and 10 mM (40.0%) did not vary significantly as compared to control seeds (43.3%) (Fig. 1a). Similar trends were observed for GRI and SVI whereby 1 mM of proline priming resulted in the highest GRI (4.5) and SVI (238.5) as compared to 2.7 (GRI) and 144.2 (SVI) in the control. In contrast, 20 mM proline significantly reduced the GRI (0.6) and SVI (63.4) as compared to other concentrations. The MGT did not vary significantly among the different proline concentrations as compared to control (Fig. 1d).

### Qualitative and Quantitative Amylase Activity

**Figure 2:**
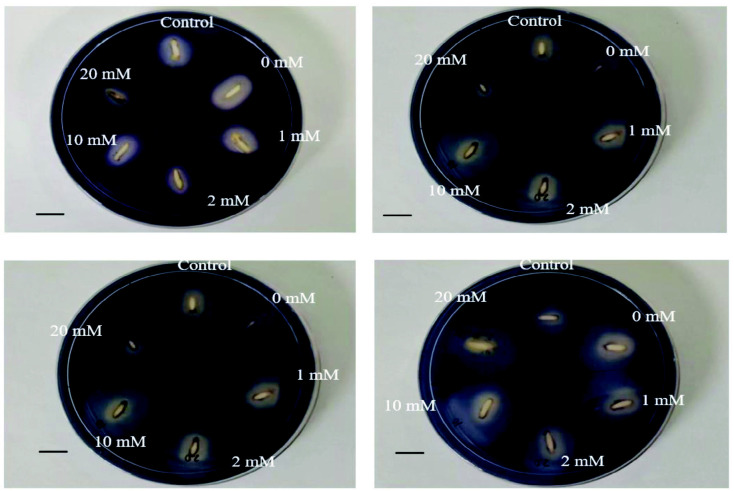
Qualitative amylase activity detected in rice seeds primed with different concentrations of proline: (a) Day 0; (b) Day 4; (c) Day 8; and (d) Day 12. *Note*: Scale bar represents 1 cm.

**Figure 3:**
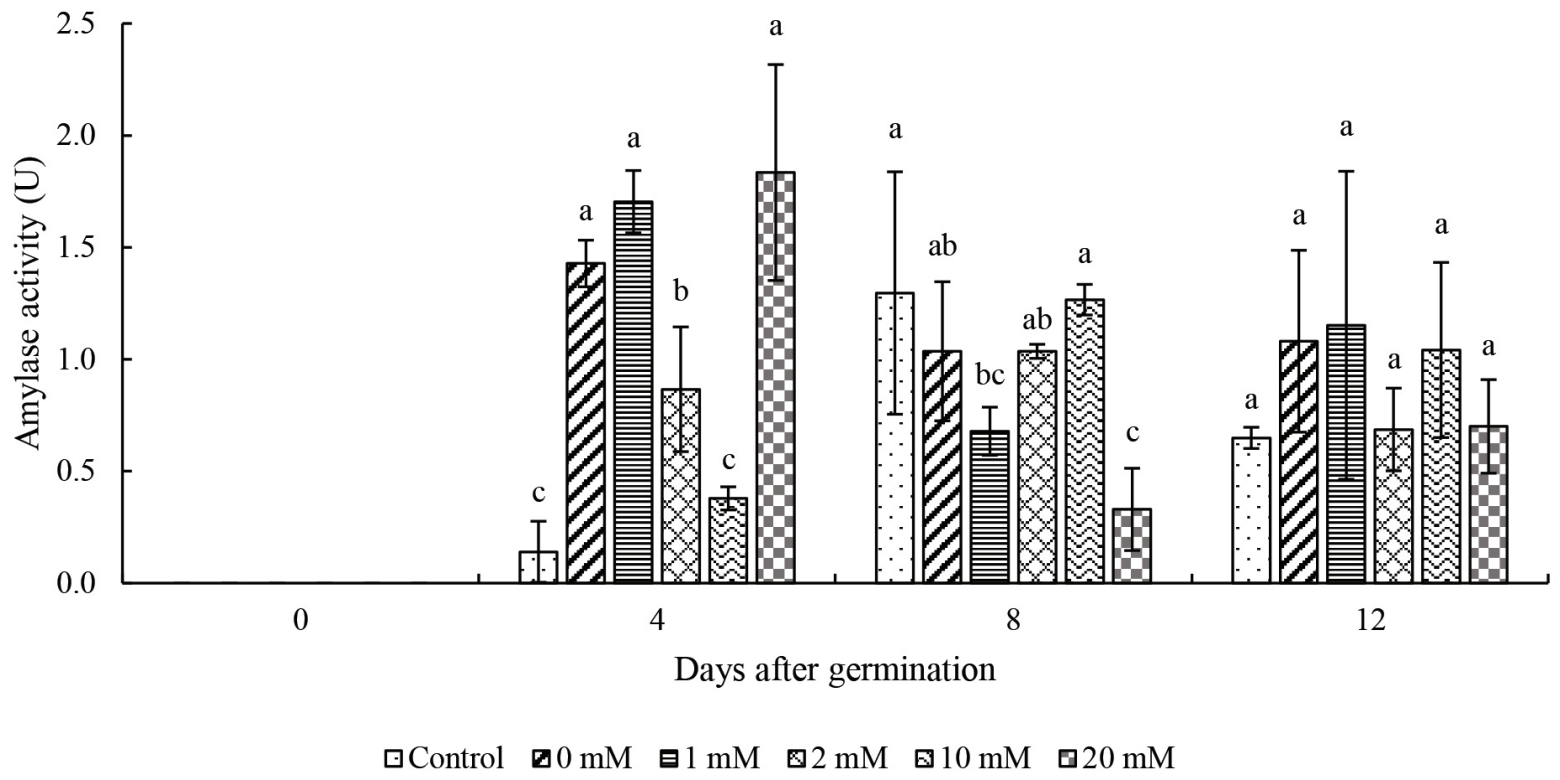
Effect of proline priming on amylase activity from Day 0 to 12. *Notes*: Bar indicates mean standard deviation of three replicates. Different lowercase letters above the bars indicate their relative significance at *p* < 0.05 probability value at different days.

Results from the qualitative plate assays showed that the clear zones were the smallest at day 0 and increase thereafter with the incubation period (Fig. 2a). At Days 4 and 8, seed primed with 10 mM proline showed the largest clear zone while 20 mM proline showed the smallest clear zone as compared to the control. The sizes of the clear zones were almost similar among the primed seed at Day 12 (Fig. 2d). As for the quantitative results, significant differences in the amylase activity were observed among the treatments at Days 4 and 8 but not for Day 12 (Fig. 3). No amylase activity was detected at Day 0. Significant increase in the amylase activity was observed starting from Day 4. Among the treatments, seeds primed with 20 mM proline treatment exhibited the highest amylase activity (1.83 U) but it is not significantly different with 0 and 1 mM proline. At Day 8, the lowest activity was recorded in 20 mM proline. At Day 12, there is no significant difference in the amylase activity among all the treatments.

### TSS Content

**Figure 4:**
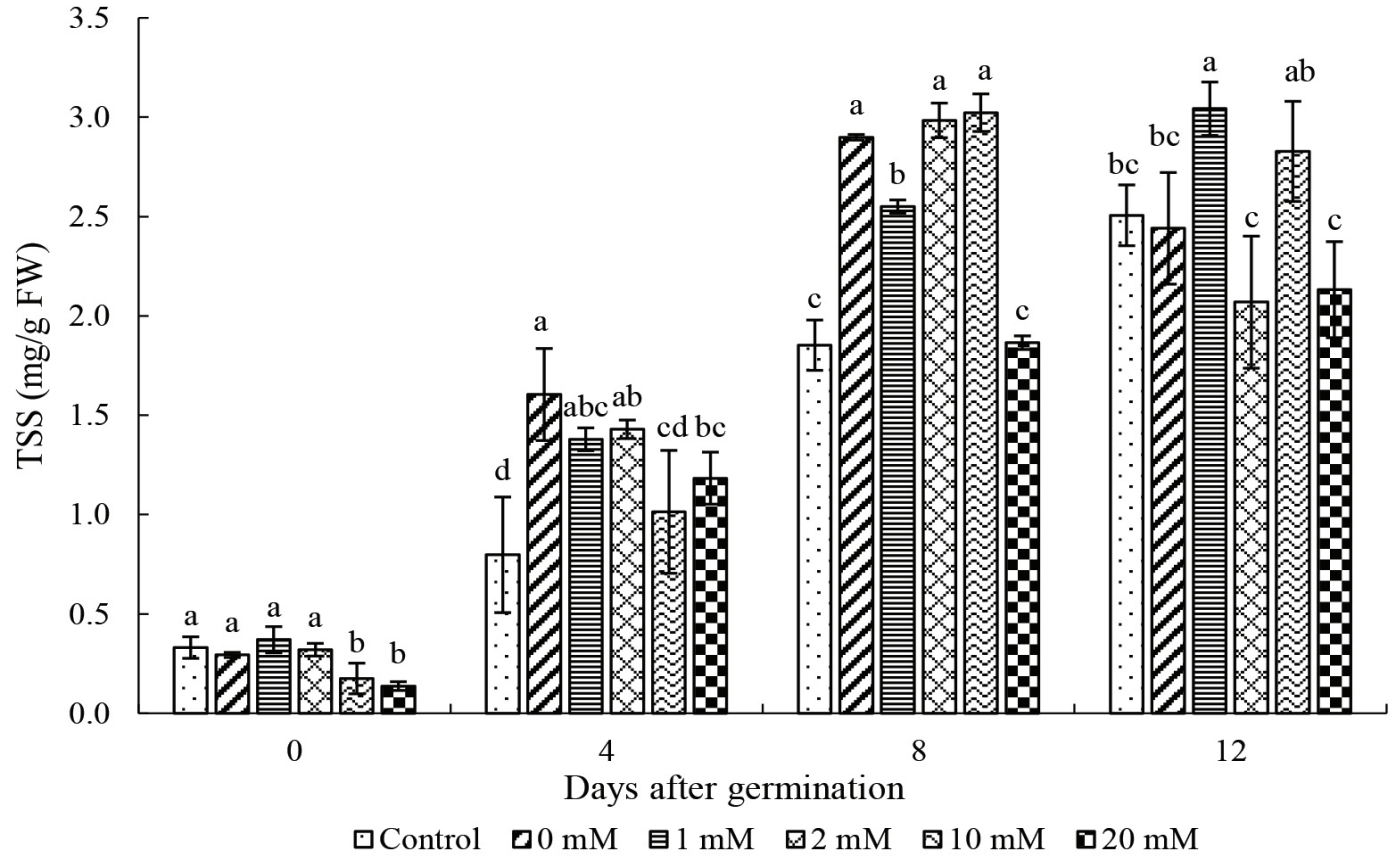
Effect of proline priming on TSS content from Day 0 to 12. *Notes*: Bar indicates mean standard deviation of three replicates. Different lowercase letters above the bars indicate their relative significance at *p* < 0.05 probability value at different days.

Results showed that the TSS content at Day 0 was the lowest compared to other days and the TSS content was higher at Days 8 and 12 as compared to Day 4 (Fig. 4). At Day 4, the TSS content in all the proline primed seeds was significantly higher (1.01–1.60 mg/g FW) than the control (0.80 mg/g FW). There is a substantial increase in the TTS content at Day 8 as compared to Day 4. At Day 8 of germination, the TSS were significantly higher with 0, 1, and 10 mM as compared to the others. At Day 12, the TSS content was significantly lower in seed primed with 2 and 20 mM than the others. Consistent increase in the TSS content from Days 0 to 12 was observed in seeds primed with 1 mM proline. In contrast, the TSS content was relatively low in seeds primed with 20 mM at different days.

## DISCUSSION

### Seed Vigour and Germination Capacity

Germination is one of the most important stages in the life cycle of various plants including rice. In this study, seeds primed with 1 mM proline resulted in higher GP, GRI and SVI among the five different proline concentrations tested. The enhancement of germination capacity of proline-primed seeds could be due the roles of proline as a potent osmoprotectant and antioxidant molecule (Ambreen *et al*. 2021). During germination, water uptake increases the turgor pressure of cells to expand and hydrate enzyme and food supplies (Makhaye *et al*. 2021). Osmoprotectants are involved in reducing harmful effects from abiotic stress by maintaining cell turgidity (Perveen & Nazir 2018). Turgid cells can increase seed germination by enhancing water uptake (Makhaye *et al*. 2021). On the other hand, seeds primed with 20 mM proline had the lowest GP, GRI and SVI but highest MGT among various proline treatments. Despite the beneficial effects of exogenous proline application, the toxic effects on plants at excessive concentrations has been reported. For instance, imbalance in inorganic ions and poor tomato growth has been observed when 40 mM of proline was applied (Heuer 2003). Also, organogenesis was inhibited in *Arabidopsis* at high concentration of proline due to the feedback inhibition of Δ^1^-pyroline-5-carboxylate synthetase (Zhang *et al*. 1995).

Similar results were reported by Deivanai *et al*. (2011) whereby rice seeds treated with low proline concentration (1 mM) was found to be effective in enhancing the plant development compared to seeds treated with high proline concentration (10 mM) under salt stress. This is because high proline in plant cells might cause negative effects on proteins functions and cell development (Dawood *et al*. 2021). However, Kamran *et al*. (2009) reported that 20 mM and 40 mM proline significantly improved the wheat growth and yield components under drought stress. Rady and Mohamed (2018) concluded that wheat showed growth improvement when primed with 10 mM–30 mM proline but inhibited when primed with proline less than 10 mM. In another report, Ambreen *et al*. (2021) reported that 15 mM of proline enhanced the growth of wheat. These results showed that the optimum level of proline is highly species dependent.

### Amylase Activity

Hydrolysis of stored food by alpha amylase is an important step during germination of rice seeds to provide energy source for the embryo to grow (Damaris *et al*. 2019). It is hypothesised that seed priming improves seed reserve mobilisation by activating or synthesising critical enzymes for germination (Langeroodi & Noora 2017). The reason of no amylase activity detected at Day 0 could be due to absence of gibberellic acid (GA) in the embryo during pre-germination of rice seeds. This is because during early seed germination, alpha-amylase present in the aleurone layer is produced in the presence of GA, which comes from the embryo (Kaneko *et al*. 2002). The active GA triggers the expression and release of amylase from the aleurone layer into endosperm to aid the process of starch degradation (Kaneko *et al*. 2002). Liu *et al*. (2018) reported that amylase activity was affected and germination was inhibited when there is insufficient active GA in rice seeds. The results obtained were similar with Saleh *et al*. (2019) whereby the amylase activity in wheat was higher during the initial phase and peaked at Day 6.

### TSS Content

Soluble sugars are produced through hydrolysis of starch by amylase enzyme and are crucial for sustaining the general structure and development of plants. It has been reported that high TSS in rice seed may cause better osmotic adjustment and maintains cell turgidity for growth (Dien *et al*. 2019). Wang *et al*. (2018) reported that salicylic acid priming significantly improved sucrose contents in wheat compared to those without priming. Similarly, selenium and salicylic priming increased the soluble sugar content in rice seeds by 21.8% and 62.7%, respectively, at 6 and 9 days after sowing (Nie *et al*. 2022). The present study’s results were in agreement with these findings whereby proline priming significantly enhanced the TSS content at Days 4 and 8 as compared to the control. The TSS content maybe different among the proline priming treatments due to the different rate of sugar utilisation. The distribution of soluble sugar between embryo and endosperm may also be different because of sugar transport and sugar metabolism between them (Wang *et al*. 2018).

## CONCLUSION

Proline priming significantly affected the seed vigour, germination capacity, amylase activity, and TSS content in rice seeds. Rice seeds primed with 1 mM proline exhibited a higher GP, SVI and GRI as compared to the control. In contrast, priming using 20 mM proline was found to be deleterious on the seed germination with the lowest GP and SVI, and higher MGT. Fluctuating trend in the amylase activity of the rice seed was observed as a result of proline priming. The TSS content of rice seeds primed with 1 mM and 20 mM of proline increased consistently from Day 0 to 12. This research demonstrated the potential of proline as a seed priming agent to enhance seed vigour and germination capacity. Future studies can be carried out to compare the changes in the expression of important genes involved in seed germination, α-amylase, soluble sugar, and proline biosynthesis to understand the mechanisms involved at the molecular level.
